# Cbx7 is epigenetically silenced in glioblastoma and inhibits cell migration by targeting YAP/TAZ-dependent transcription

**DOI:** 10.1038/srep27753

**Published:** 2016-06-13

**Authors:** Zahid Nawaz, Vikas Patil, Anjali Arora, Alangar S. Hegde, Arimappamagan Arivazhagan, Vani Santosh, Kumaravel Somasundaram

**Affiliations:** 1 Department of Microbiology and Cell Biology, Indian Institute of Science, Bangalore 560012, India; 2 Department of Neurosurgery, Sri Satya Sai Institute of Higher Medical Sciences, Bangalore 560066, India; 3 Departments of Neurosurgery, National Institute of Mental Health and Neuro Sciences, Bangalore 560029, India; 4 Department of Neuropathology, National Institute of Mental Health and Neuro Sciences, Bangalore 560029, India.

## Abstract

Glioblastomas (GBM) are the most malignant form of astrocytomas which are difficult to treat and portend a grave clinical course and poor prognosis. In this study, we identified Chromobox homolog 7 (Cbx7), a member of Polycomb Repressive Complex 1 (PRC1), as a downregulated gene in GBM owing to its promoter hypermethylation. Bisulphite sequencing and methylation inhibitor treatment established the hypermethylation of Cbx7 in GBM. Exogenous overexpression of Cbx7 induced cell death, inhibited cell proliferation, colony formation and migration/invasion of the glioma cells. GSEA of Cbx7 regulated genes identified Cbx7 as a repressor of transcription co-activators YAP/TAZ, the inhibitory targets of the Hippo signalling pathway. In good correlation, the exogenous expression of Cbx7 repressed the YAP/TAZ-dependent transcription and downregulated CTGF, a bonafide YAP/TAZ target. We also observed reduced levels of phospho-JNK in Cbx7 expressing cells. Additionally, CTGF silencing and pharmacological inhibition of JNK also inhibited glioma cell migration. Further, Cbx7 failed to inhibit cell migration significantly in the presence of exogenously overexpressed CTGF or constitutively active JNK. Thus, our study identifies Cbx7 as an inhibitor of glioma cell migration through its inhibitory effect on YAP/TAZ-CTGF-JNK signalling axis and underscores the importance of epigenetic inactivation of Cbx7 in gliomagenesis.

Cancer involves sequential accumulation of changes in a cell which potentiate it to become malignant or increase its severity of malignancy; hence it is often thought to be progressive in nature. After the first few catastrophic changes that get imbued in the genome, the passage of time incorporates a plethora of detrimental changes in a cell and subsequently brings it to a state from where there is no retreat. Though very meagre is known about the sequence in which these changes produce a malignant phenotype but the nature of these alterations is quite well understood. All these changes pave way for a tumour cell to surmount anti-proliferative signals and gain growth factor independence, ultimately leading to its superior survival. While most of these alterations in the genome comprise of discrete genetic events such as copy number aberrations, mutations and gene translocations; epigenetics events have also gained an acceptable recognition on this platform. Epigenetic alterations broadly constitute of all those special chemical marks on DNA and histones that collectively determine whether a gene is accessible to transcription^1^. These changes are predominantly of two types. DNA methylation, which involves the methylation of specific Cytosine residues immediately followed by Guanidine i.e. CpG, and when many such events happen in a close vicinity, it results in the transcriptional shutdown of that locus. The other type comprises of various kinds of Histone modifications in terms of methylations, acetylations and ubiquitinations.

Glioblastoma (GBM) is the most common subtype of gliomas which account for about 80% of primary brain tumours^2^,^3^. Malignant gliomas are difficult to treat and portend a grave clinical course and poor prognosis^4^. Inspite of all therapeutic modalities the median survival of GBM is around 12–15 months^5^. Current treatment regimens comprise of tumour resection followed by radiation and concomitant chemotherapy, but inspite of all this improvement, better survival is still awaited. Although a lot has been uncovered and deciphered about the alterations in GBM at the genetic level, epigenetic abnormalities need to be comprehended extensively. These epigenetic modifications, which are commonplace in GBM, necessitate urgent consideration for the better understanding of the malignancy.

Polycomb proteins are a group of proteins which facilitate a class of epigenetic events in a cell and add yet another realm of regulation in gene expression. Polycomb group of proteins are classified into two multi-protein complexes: Polycomb repressive complex 1, PRC1 and Polycomb repressive complex 2, PRC2^6^. The PRC2 protein complex which comprises of Enhancer of Zeste (EZH2), Early embryonic deficient (EED), Suppressor of Zeste (SUZ12) and other associated proteins conduct histone de-acetylation and histone methylation, specific to the lysine 27 of histone 3, thereby leaving a transcriptionally repressive mark on the chromatin^6^. Such alterations are transcriptionally repressive and are identified and read by PRC1 protein complex which comprises of the mammalian homologs of Drosophila Polycomb (Pc), Posterior sex combs (Psc), Sex combs extra (Sce) and Polyhomeiotic (Ph)^6^. This sequential feat by PRC2 followed by PRC1 induces further chromatin remodelling and ultimately transcriptional shut down of the locus. One of the important components of the PRC1 is the polycomb protein (Pc) known as chromobox protein in humans and other mammals^7^. Chromobox (Cbx) proteins are called so, owing to the presence of chromodomain motif (*chromatin organisation modifier*) in their structure. There are five chromobox proteins in humans, Cbx2, 4, 6, 7 and 8 and the pattern of their expression varies spatio-temporally^7^. Like other members of the PRC1 and PRC2 complexes, a number of studies and approaches have unveiled the role of Cbx proteins in tumorigenesis. For instance, Cbx4 plays a crucial role in hepatocellular carcinoma by potentiating HIF1-alpha and bolstering the expression of VEGF^7^. Cbx8 knock-down exerts a paradoxical role in the progression of colorectal cancer by inhibiting proliferation while stimulating metastasis by enhancing migration and invasion capacities of tumour cells^8^. Cbx6 overexpression leads to reduced proliferative capacity of glioma cells^9^. Cbx7, yet another Chromobox protein, behaves rather ambiguously in different human malignancies owing to its dual role, both as an oncogene as well as a tumour suppressor. Cbx7 works more like a growth promoting factor, depending upon the cellular state, as in the case of primary cells it increases the lifespan of primary human prostate epithelial cells^10^. Cbx7 is also reported to immortalize mouse embryonic fibroblasts (MEF) by repressing the INK4a/ARF locus^10^. Cbx7 functions as an oncogene by producing aggressive B-cell lymphomas^11^. On the other hand, Cbx7 gene is heavily downregulated in thyroid carcinomas as well as in pancreatic cancer^12^. This made us to elucidate the potential contribution of Cbx7 in glioblastoma biology.

## Results

### Cbx7 is epigenetically silenced in malignant glioma

**Figure 1:**
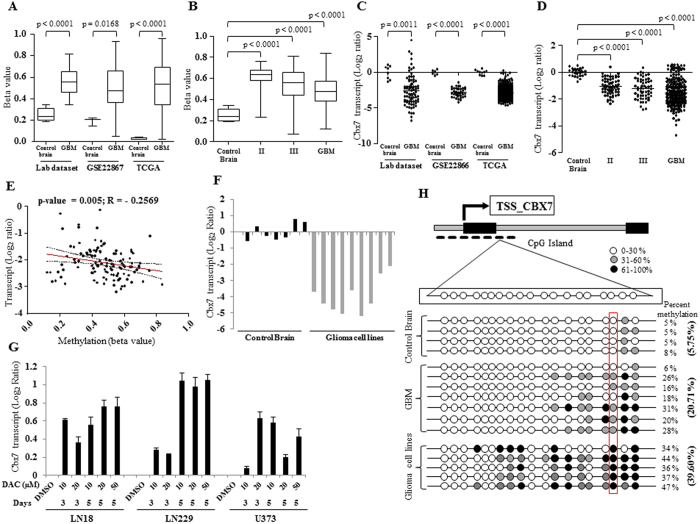
Cbx7 is methylated and downregulated in GBM. (**A**) Beta values of Cbx7 in control brain (lab dataset n = 9, GSE28867 dataset n = 3, lab dataset n = 8), Glioblastoma samples (lab dataset n = 36, GSE28867 dataset n = 55, TCGA dataset n = 295), plotted as box and whisker plots. t-test was performed between control brain and GBM samples, *p* values are indicated. (**B**) Beta values of Cbx7 in control brain (n = 9) (lab dataset) and different glioma grades; (Grade II n = 63, Grade III n = 131, GBM n = 143), from TCGA data set, plotted as box and whisker plot. Significance testing was performed using ANOVA (Tukey, post hoc) across different sample groups, overall *p* value was < 0.0001 and the comparative *p* values are indicated. (**C**) Cbx7 transcript levels derived from various datasets in control brain (lab dataset n = 8, GSE28866 dataset n = 6, TCGA dataset n = 10) and Glioblastoma samples (lab dataset n = 82, GSE28866 dataset n = 40, TCGA dataset n = 548), were plotted as scatter plot. t-test was performed between control brain and GBM samples, *p* values are indicated. (**D**) Cbx7 transcript levels across different glioma grades (REMBRANDT data set) (control brain n = 28, Grade II n = 65, Grade III n = 58, GBM n = 227), plotted as scatter plot. Significance testing was performed using ANOVA (Tukey, post hoc) across different sample groups, overall *p* value was < 0.0001 and the comparative *p* values are indicated. (**E**) Correlation curve graph, depicting significant negative correlation between Cbx7 methylation and its expression in TCGA dataset; *p* value and correlation coefficient (R) are indicated. (**F**) Cbx7 transcript levels in control brain and various glioma cell lines (U87, U138, U251, U343, U373, LN18, LN229, and SVG) and HEK293T using qRT-PCR is plotted. (**G**) RNA was isolated from LN18, LN229 and U373 after treatment with 5-aza-2-deoxycytidine (DAC) for 3 and 5 days with 10, 20 and 50 μM concentrations. Cbx7 transcript levels were quantified by qRT-PCR. (**H**) Bisulphite sequencing of Cbx7 promoter region. Each row indicates methylation pattern of one sample. Percentage methylation of promoter was calculated as total percent of methylated cytosines from 10 randomly sequenced colonies. TSS, transcription start site, dashed line represents the CpG island analyzed by sequencing, red box represents CpG corresponding to cg23124451 probe in the Infinium array.

Genome-wide methylation analysis that was carried out using Infinium HumanMethylation450K BeadChip array, revealed Cbx7 as one of the hypermethylated genes in GBM (Fig. 1A) and lower grades of glioma (Fig. 1B) compared to control brain samples in our cohort, GSE 228867 and TCGA data sets. Cbx7 belongs to the chromobox family of proteins which are characterized by the presence of chromodomain. While Cbx1, Cbx3 and Cbx5 are known as heterochromatin proteins 1β, γ and α respectively (HP1β, γ and α), Cbx2, Cbx4, Cbx6, Cbx7 and Cbx8 form components of Polycomb repressive complex 1 (PRC1). PRC1 and PRC2 complexes form the family of transcriptional repressors under polycomb group (PcG) proteins that play a key role in development and pluripotency. Investigation of TCGA (The Cancer Genome Atlas) data set revealed that while Cbx1 and Cbx5 are not regulated, Cbx3 is upregulated in GBM compared to control brain samples, although not due to differential methylation (Supplementary Figure S1A and S1C). Among Cbx proteins that belong to PRC1 complex, Cbx6 and Cbx7 are downregulated, while others are not regulated in GBM compared to control brain samples (Supplementary Figure S1B). While Cbx7 downregulation is due to promoter hypermethylation (Supplementary Figure S1D; Fig. 1), the mechanism behind Cbx6 downregulation is not known at present. We have concentrated our efforts on Cbx7 regulation and its functions in the context of glioma.

We observed a significant downregulation of Cbx7 transcript levels in GBM (Fig. 1C) and low grade gliomas (Fig. 1D) compared to control brain samples in our cohort, GSE 228866 and TCGA data sets. We found a significant negative correlation between Cbx7 transcript level and it’s promoter methylation (Fig. 1E). We also found that Cbx7 was downregulated in glioma cell lines compared to the control brain samples (Fig. 1F) and the treatment with methylation inhibitor resulted in the re-expression of Cbx7 transcript in three different glioma cell lines (Fig. 1G). Further, bisulphite sequencing confirmed the hypermethylation of Cbx7 promoter in GBM samples (20.71%), glioma derived cell lines (39.60%) as compared to control brain samples (5.75%) (Fig. 1H). Thus from these results, we conclude that Cbx7 is a silenced gene in GBM on account of its promoter hypermethylation.

### Cbx7 inhibits glioma cell growth and migration

**Figure 2:**
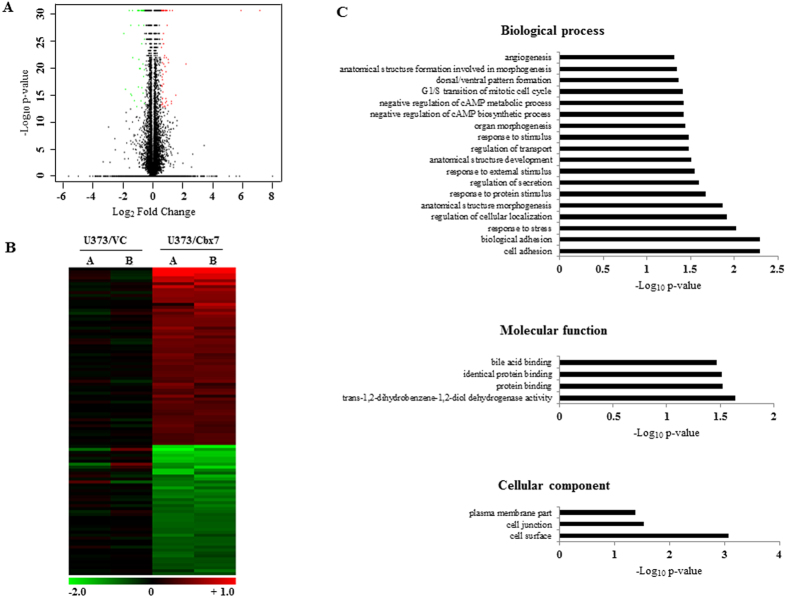
Cbx7 mediates changes in gene expression, implicating its role in cell growth, migration and invasion. (**A**) Volcano plot representation of the RNA sequencing data, indicating the genes significantly and differentially expressed between Cbx7 overexpression and vector control condition, each red dot indicates a gene significantly upregulated in Cbx7 overexpression condition and each green dot indicates a gene significantly downregulated in Cbx7 overexpression condition. The x-axis shows the log_2_ fold FPKM change (Fragments Per Kilobase of transcript per Million mapped reads) and the y-axis shows the *p* value expressed in −log_10_ scale. (**B**) Heat map indicating the shortlisted genes which are significantly upregulated and downregulated in Cbx7 overexpression state with a cut-off of +/−0.57 in the log_2_ scale over the control vector. (**A,B**) represent the duplicate runs of each sample. (**C**) Gene ontology analysis indicating the different processes enriched for the significantly altered genes in Cbx7 overexpression state.

To further understand the necessity for Cbx7 silencing by promoter methylation in glioma, we carried out transcriptome profiling of U373 cell line transfected with control vector (U373/VC) or Cbx7 cDNA (U373/Cbx7) by whole RNA sequencing. Unbiased functional enrichment analysis using the significantly differentially regulated genes in Cbx7 overexpressed condition (Fig. 2A,B; Supplementary Table ST1) revealed an enrichment of various gene ontology terms like cell adhesion, biological adhesion, stress response, cell cycle, morphogenesis and structure, cell junction and cell surface, which cumulatively hinted to the role of Cbx7 in modulating the migratory capacity, cell growth and cell cycle like phenotypes (Fig. 2C; Supplementary Table ST2).

**Figure 3:**
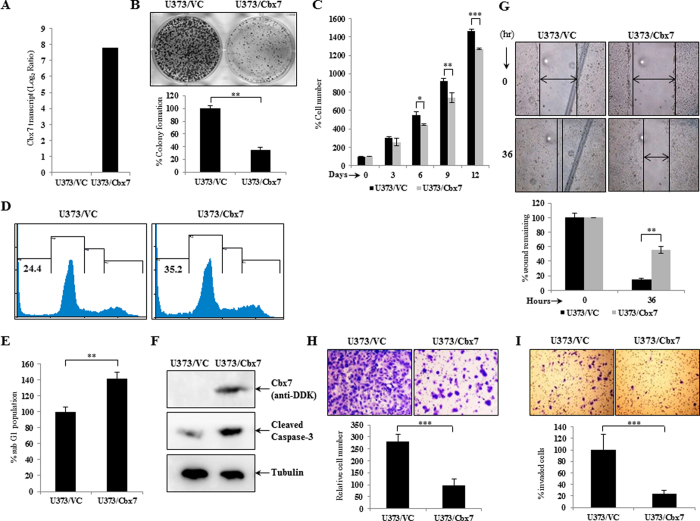
Cbx7 affects the different tumorigenic properties of glioma cells. (**A**) U373 cell line was transfected with pCMV-Vector and pCMV-Cbx7 and selected for G418 resistance for two weeks. The stable U373/VC and U373/Cbx7 were harvested for RNA and protein extraction. The column plot indicates Cbx7 transcript levels, as assayed by qRT-PCR. (**B**) U373/VC and U373/Cbx7 stables were counted and plated at a density of 2.5 × 10^3^ cells per well and allowed for colony formation. Colonies were stained with crystal violet. Mean colony counts are displayed as percentage of vector control. (**C**) Control Vector and Cbx7 overexpressing stables of U373 cell line were counted and plated (4.0 × 10^4^ cells) for proliferation in duplicates. Cells were counted every third day using viability cell counter. The column plot indicates the percent cell number at the respective time intervals. (**D,E**) U373 cell line was transfected with pCMV-Vector and pCMV-Cbx7, after 48 hours cells were harvested for cell cycle analysis by propidium iodide staining, the numerals (bold) represent the percent sub-G1 population (apoptotic cells). The column plot depicts the quantitation of sub-G1 cell population from three independent experiments. Percent sub-G1 population with standard deviation is plotted. (**F**) The western blot indicates more of cleaved caspase-3 in Cbx7 overexpression condition, indicating more apoptosis. (**G**) U373/VC and U373/Cbx7 stable cells were subjected to *in vitro* scratch assay, the area of scratch was photographed at the time of scratch (0^th^ hour) and after regular intervals to monitor wound closure. The column plot represents the gap of the wound (average of 8 different locations from two different wells). (**H,I**) U373/VC and U373/Cbx7 stable cells were plated for Boyden chamber migration assay and matrigel invasion assay. After 22 hours, cells were fixed, stained with crystal violet and photographed. The column plot represents the number of cells migrating or invading respectively, cell number with standard deviation is plotted. Significance testing was performed between U373/VC and U373/Cbx7 in different experiments using student’s t-test and the symbols are indicated. *p ≤ 0.05; **p ≤ 0.01 and ***p ≤ 0.001.

To assess the effect of Cbx7 on glioma cell lines, we performed a transient transfection of Cbx7 in various glioma cell lines and assayed for certain parameters. Cbx7 overexpression inhibited colony formation (Supplementary Figure S2A–C) indicating its role in growth suppression. U373 cells stably overexpressing Cbx7 (U373/Cbx7) (Fig. 3A) also exhibited drastic reduction in growth as evidenced by colony suppression and reduced proliferation of glioma cells as compared to the vector control (U373/VC) (Fig. 3B,C). Over-expression of Cbx7 also resulted in increased sub-G1 cell population indicating more apoptosis (Fig. 3D,E). This was further corroborated by more levels of cleaved caspase-3 in U373/Cbx7 cells (Fig. 3F). U373/Cbx7 stable cells also showed decreased migration and invasion as elucidated through scratch assay and Boyden chamber transwell migration and invasion assays (Fig. 3G–I).

### Cbx7 activates Hippo pathway by inhibiting YAP/TAZ-dependent transcription

**Figure 4:**
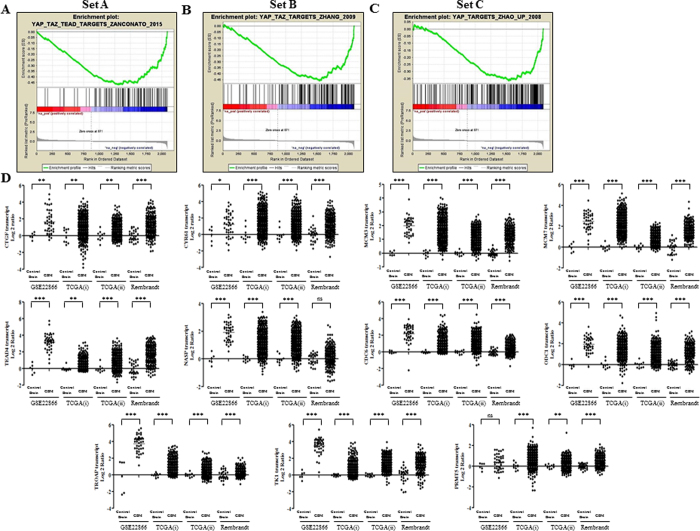
Cbx7 synergizes the hippo signalling pathway activity. (**A–C**) Gene Set Enrichment Analysis (GSEA) enrichment plots of the Cbx7 regulated genes among the three sets of YAP/TAZ targets indicating significant negative enrichment. (**D**) Majority of the important YAP/TAZ target genes negatively enriched upon Cbx7 overexpression were found to be upregulated in GBM across different data sets [GSE22866, TCGA(i)-Affymetrix, TCGA(ii)-Agilent and Rembrandt]. Significance testing was performed between control brain and GBM samples using student’s t-test and the symbols are indicated, (ns) not significant; *p ≤ 0.05; **p ≤ 0.01 and ***p ≤ 0.001

Next, we investigated the possibility of crosstalk between Cbx7 and Hippo signalling pathway as the latter pathway is well known for its role in cell proliferation, apoptosis, metastasis and invasion of cancer cells^13^, Gene set enrichment analysis (GSEA) revealed a significant negative enrichment of Yes-associated protein (YAP)/Tafazzin (TAZ) target genes (identified by three independent studies^14^,^15^,^16^; Supplementary Table ST3–5) in Cbx7 overexpressed condition (Fig. 4A–C). We also found that the majority of enriched YAP/TAZ target genes that were common to all three gene sets, to be upregulated in GBM compared to control brain samples in multiple data sets (Fig. 4D; Supplementary Table ST6–8). This indicates that Cbx7 synergizes the effects of Hippo pathway by impeding the transcription mediated by YAP/TAZ.

**Figure 5:**
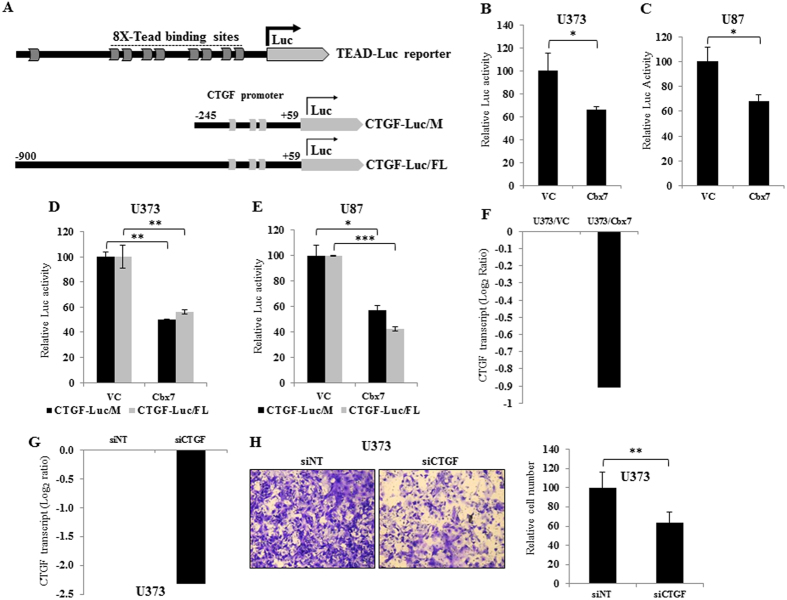
Cbx7 abrogates YAP/TAZ dependent transcription and downregulates the expression of CTGF to bring about the reduction in migration. (**A**) Pictorial representation of the TEAD-Luc reporter, the construct has 8-TEAD binding sites in tandem, upstream to the luciferase gene. The reporter is responsive to the changes in transcription driven by YAP/TAZ and TEAD transcription factor. Pictorial representation of the CTGF promoter reporter (below the TEAD-Luc reporter); minimal promoter (CTGF-Luc/M) and the full length promoter (CTGF-Luc/FL) were synthesized by cloning the upstream promoter region of CTGF as indicated. Both the promoter reporters have three canonical TEAD binding sites indicated as grey bars. (**B**,**C**) Cbx7 vector or control vector was transfected along with TEAD-luc luciferase reporter in U373 and U87cells, after 48 hours, luciferase activity was measured and plotted. (**D**,**E**) Cbx7 vector or control vector was transfected along with minimal or full length CTGF promoter reporter in U87 and U373 cell lines. After 48 hours, luciferase activity was measured and plotted. (**F**) CTGF transcript level in U373/Cbx7 cells as compared to U373/VC cells; y-axis represents the transcript levels in Log_2_ scale. (**G**) CTGF transcript levels after siRNA treatment in U373 cell line, y-axis represents the Log_2_ fold change as compared to siRNA non targeting control (siNT). (**H**) U373 cells transfected with siNT and siCTGF were counted and plated for Boyden Chamber migration assay. After 22 hours, cells were fixed, stained with crystal violet, and photographed. The column plot represents the relative cell number capable of migrating through, percent cell number with standard deviation is plotted. Significance testing was performed using student’s t-test and the symbols are indicated, *p ≤ 0.05; **p ≤ 0.01 and ***p ≤ 0.001.

Next to confirm this hypothesis, we experimentally validated the inhibitory effect of Cbx7 on YAP/TAZ-dependent transcription. Exogenous expression of Cbx7 reduced YAP/TAZ-dependent transcription from TEAD-Luc reporter (a luciferase promoter reporter responsive to TEAD protein, to which YAP/YAZ bind to bring about transcriptional activation) (Fig. 5A) in U373 and U87 cells (Fig. 5B,C respectively). Further, we concentrated our efforts on the Cbx7 regulation of CTGF (***C***onnective ***t***issue ***g***rowth ***f***actor), a bonafide target of YAP/TAZ which is known to mediate cell migration^17^,^18^ and is also upregulated in glioma (Fig. 4D). Exogenous expression of Cbx7 abrogated transcription from CTGF promoter driven luciferase construct (Fig. 5A) in U373 and U87 cells (Fig. 5D,E respectively). U373/Cbx7 stable cells had reduced CTGF transcript levels (Fig. 5F). Silencing of CTGF by siRNA (Fig. 5G) reduced the migration capacity of U373 cells (Fig. 5H). From these results, we conclude that Cbx7 activates Hippo pathway by inhibiting YAP/TAZ-dependent transcription, in particular of CTGF.

### Cbx7 overexpression leads to SAPK/JNK activity loss, thereby loss of migration of tumour cells

**Figure 6:**
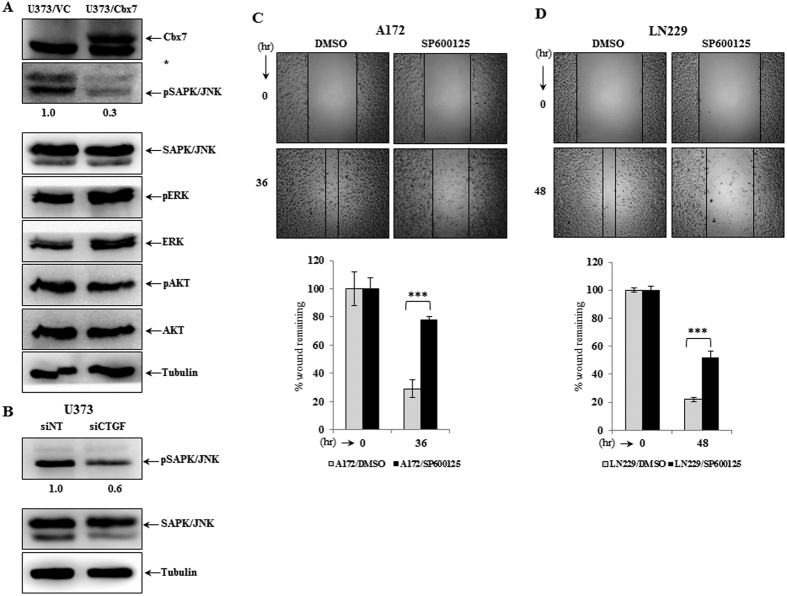
SAPK/JNK is the effector molecule responsible for Cbx7 mediated delayed migration. (**A**) Total cellular protein was extracted from U373/VC and U373/Cbx7 and the expression level of indicated proteins was assayed using Western blot, asterisk (*) refers to a non-specific band obtained with anti-Cbx7 antibody. All PAGE gels were run under same experimental conditions. (**B**) U373 cells were treated with siNT (siRNA non targeting) and siCTGF, after 72 hours protein was extracted to assay the levels of phospho-SAPK/JNK. (**C**,**D**) A172 and LN229 cell lines were treated with SP600125 (50 μM) (SAPK inhibitor) followed by *in vitro* scratch assay, the scratch area were photographed at 0^th^ hour and thereafter at regular intervals. The bar graphs represent the gap of the wound (average of 8 different locations from two wells with standard deviation) at the beginning and at the end of the experiment. Significance testing was performed using student’s t-test and the symbols are indicated, ***p ≤ 0.001.

To dissect the signalling pathway downstream of CTGF responsible for Cbx7 mediated inhibition of glioma cell migration, we analysed the phosphorylation status of various kinases in Cbx7 overexpressing cells. Cbx7 overexpression resulted in substantial reduction in phospho-JNK (c-Jun N-terminal kinase), a stress activated protein kinase, but failed to have any effect on phospho-ERK and phospho-AKT (Fig. 6A and Supplementary Figure S4A). CTGF silencing also resulted in reduced phospho-JNK levels (Fig. 6B). Direct inhibition of JNK by a pharmacological inhibitor SP600125 also resulted in reduced glioma cell migration (Fig. 6C,D and Supplementary Figure S4B,C). All together, we conclude from these results that Cbx7 inhibits glioma cell migration by inhibiting YAP/TAZ-dependant activation of CTGF with the resultant reduced signalling by phospho-SAPK/JNK.

### Inhibition of CTGF-pSAPK/JNK cascade by Cbx7 is essential for its anti-migratory phenotype

**Figure 7:**
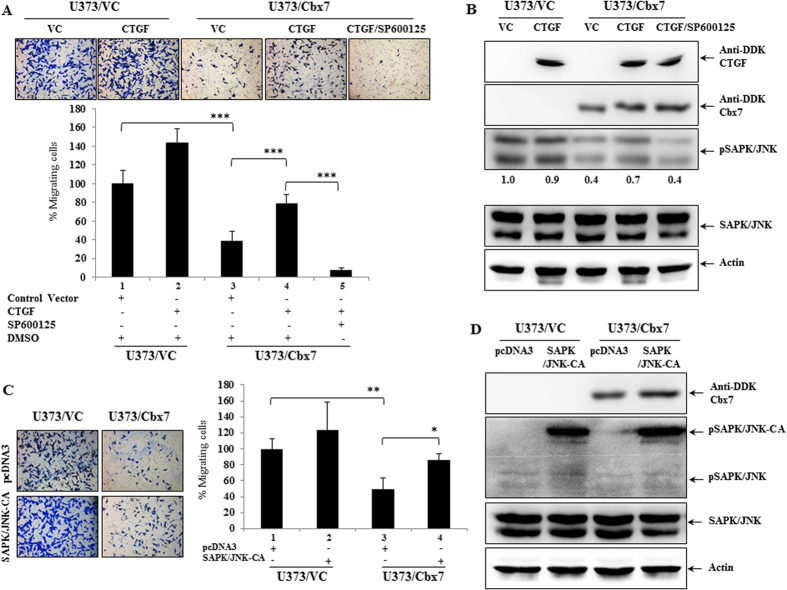
CTGF re-expression and SAPK/JNK activation rescues the migratory potential of glioma cells. (**A**) U373/VC and U373/Cbx7 stable cells were transfected with CTGF expression construct and control vector. After 48 hours of transfection, cells were plated for Boyden chamber migration assay. 22 hours later, cells were fixed, stained with crystal violet, and photographed. Under similar conditions, U373/Cbx7 cells transfected with CTGF were also treated with SP600125 (50 μM) (SAPK inhibitor). The column plot represents the relative number of cells capable of migrating, percent cell number with the standard deviation is plotted. Significance testing was performed using ANOVA (Bonferroni, post hoc) across different experimental conditions, overall *p* value was < 0.0001 and the comparative *p* values are indicated. (**B**) Total cellular protein was extracted from U373/VC and U373/Cbx7 cells transfected with CTGF and control vector, and SP600125 treated cells, as indicated in the figure. The expression level of indicated proteins was assayed using western blotting. Being a secretory protein, CTGF expression was assayed by concentrating the conditioned media and loading equal volumes of the same in a polyacrylamide gel. All PAGE gels were run under same experimental conditions. (**C**) U373/VC and U373/Cbx7 stable cells were transfected with SAPK/JNK-CA (pcDNA3-Myc-SAPKβ-MKK7) expression construct and control vector (pcDNA3). After 48 hours of transfection, cells were plated for Boyden chamber migration assay. 22 hours later, cells were fixed, stained with crystal violet, and photographed. The bar graph represents the relative number of cells capable of migrating, Percent cell number with standard deviation is plotted. Significance testing was performed using ANOVA (Bonferroni, post hoc) across different experimental conditions, overall *p* value was < 0.0001 and the comparative *p* values are indicated. (**D**) Total cellular protein was extracted from U373/VC and U373/Cbx7 cells transfected with SAPK/JNK-CA (pcDNA3-Myc-SAPKβ-MKK7) and control vector (pcDNA3) as indicated in the figure. The expression level of indicated proteins was assayed using Western blotting.

Exogenous overexpression of Cbx7 leads to reduced expression of CTGF and thereby reduced migration. To further consolidate this axis of regulation, we checked the ability of exogenously overexpressed CTGF to reverse the inhibition of migration brought about by Cbx7. We found that the exogenous overexpression of CTGF significantly rescued the migratory potential of the Cbx7 stable glioma cells (Fig. 7A, compare 3 and 4). Under similar conditions, the rescue brought about by CTGF overexpression was significantly abolished by JNK pathway inhibition (Fig. 7A, compare 4 and 5). Moreover, exogenous overexpression of CTGF also significantly increased the pSAPK/JNK levels in Cbx7 stable glioma cells (Fig. 7B). Furthermore, the overexpression of a constitutively active form of SAPK/JNK in the Cbx7 over-expression background also resulted in the retrieval of the phenotype, thereby underscoring the importance and indispensability of JNK/SAPK in the regulatory axis (Fig. 7C,D).

## Discussion

Polycomb group of proteins have been implicated in a myriad of cellular processes and phenomena including differentiation of stem cells^19^, cell cycle control^20^, cellular senescence^21^, DNA replication^22^, cell fate decisions^23^, X-chromosome inactivation^24^, maintenance of cytoskeleton^25^ and various pathogenic states like cancer^23^,^26^,^27^,^28^,^29^. In this study, we elucidated the role of Cbx7 in gliomagenesis. Cbx7 plays a key role in the functional organisation of the PRC1^30^,^31^, thereby affecting the transcription of a wide range of genes which directly or indirectly impact tumourigenesis^32^. The levels of Cbx7 inside the cell are very crucial as it is needed in precise stoichiometry necessary for the maintenance and functioning of PRC1 and PRC2 complex^31^. Any alteration in the same, results in the dysfunction of the complex, leading to the aberrant silencing of target genes, eventually resulting in various malignant neoplasias^30^,^31^. There exists a nice correlation between the loss of expression of Cbx7 and the aggressiveness of various cancers^33^,^34^. This observation is further substantiated by the fact that Cbx7 is either undetectable or very lowly expressed in most of the human cancers with a few exceptions^10^,^35^,^36^,^37^. However, the role of Cbx7 in human malignancies still remains ambiguous, owing to the fact that there are reports which portray Cbx7 as an oncogene as well as some of them depict its role as a tumour suppressor. The aberrant behaviour of Cbx7 could be possibly attributed to the cellular context or the type of interacting partners it is involved with, spatiotemporally^38^. Cbx7 was observed to be downregulated in urothelial carcinomas with its expression being least in the most aggressive cases^34^. Similarly its expression was observed to be heavily lost in thyroid, colorectal, breast, pancreas and lung carcinomas^12^,^33^,^39^,^40^,^41^,^42^. On the other hand, Cbx7 plays a decisive role in the development of B-cell lymphomas as well as in increasing the life span of MEFs and human prostrate primary epithelial cells, which indicate its role as an oncogene^10^,^11^,^36^.

In glioblastoma, a vast majority of PRC1 and PRC2 members are misregulated is a known fact and contribute to gliomagenesis in a multitude of ways like potentiating glioma stem cells, imparting radio-resistance and modulating various pro-oncogenic pathways^43^,^44^,^45^. In a recent study, Cbx6, a chromobox protein, was found to be downregulated in GBM and to have tumour suppressive effects, although its mechanism of downregulation is not understood^9^. In our study, we demonstrate that Cbx7, yet another polycomb protein is downregulated in glioblastoma and moreover, we prove that the downregulation of Cbx7 is attributed to its promoter hypermethylation. Further, we observed that the exogenous overexpression of Cbx7 leads to the suppression of colony formation, induction of apoptosis as well as loss of migratory and invasive potential of glioma cells. We also unravelled a link between Cbx7 and hippo signalling pathway, wherein we establish its role as a positive regulator of the hippo pathway.

Hippo pathway is a vastly conserved pathway which controls cell fate and tissue growth under normal cellular homeostasis, but the pathway is frequently perturbed in a broad range of human carcinomas which include lung, colorectal, ovarian and liver cancer^13^,^46^. Hippo signalling is potentiated by a core kinase cassette constituted by a pair of related serine threonine kinases: mammalian STE20-like protein kinase 1 and 2 (MST1/2) and large tumour suppressor 1 and 2 (LATS1/2)^47^,^48^. These kinases together with the adaptor proteins Salvador homologue 1 (SAV1) and MOB kinase activator 1A (MOB1A) and MOB1B bring about the phosphorylation of oncoproteins YAP and TAZ, transcriptional co-activators with PDZ-binding motif^13^,^47^,^49^. The phosphorylation tag on YAP and TAZ creates 14-3-3 binding sites which sequesters them in the cytoplasm and eventually stimulates proteolysis by Ubiquitin Proteosomal Pathway (UPP)^50^. In other case, which is prevalent in most cancers, YAP and TAZ positively regulate the activity of transcription factors like TEADs and SMADs to command tissue growth and cell viability^51^. We demonstrated that Cbx7 potentiation inside the glioma cells resulted in the loss of YAP/TAZ driven transcriptome as evidenced by the negative enrichment of the YAP/TAZ targets. Same finding was further substantiated by the loss of expression of CTGF, a bonafide YAP/TAZ target, upon Cbx7 overexpression.

**Figure 8:**
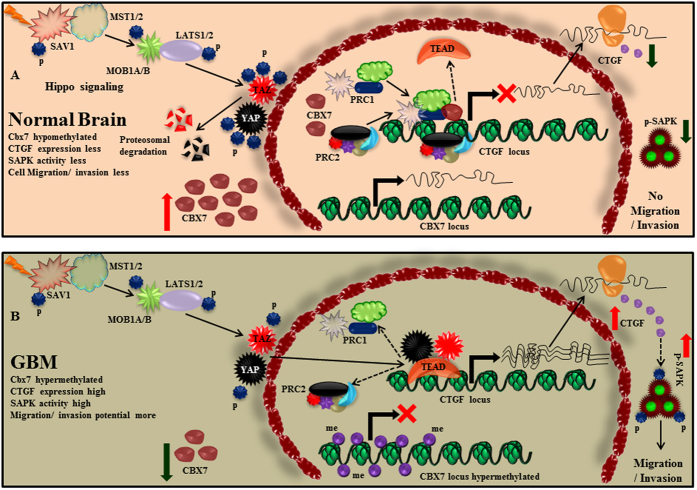
Mechanistic model for Cbx7 regulation of glioma cell migration. (**A**) Under non-malignant conditions CBX7 is not hypermethylated resulting in its adequate levels inside the cell and its proper incorporation into the PRC1 complex. This results in the transcriptional repression of a wide range of oncogenes, like CTGF (bonafide target of YAP/TAZ). (**B**) In a malignant cell CBX7 locus becomes hypermethylated and Cbx7 fails to constitute the PRC1 complex efficiently. This renders the PRC1 complex significantly non-functional which subsequently results in the upregulation of YAP/TAZ transcriptional targets like CTGF. Being a secretory molecule, CTGF executes its function by inciting various oncogenic signalling pathways on the membrane, resulting in the activation of kinases like SAPK/JNK and eventually leading to more migration.

CTGF belongs to a class of growth factors known as CCN gene family, [cysteine-rich 61 (Cyr61), connective tissue growth factor (CTGF), nephroblastoma overexpressed (Nov)]. This family consists of 6 distinct members: CYR61 (CCN1), CTGF (CCN2), NOV (CCN3), WISP-1(wnt-1-inducible gene, CCN4), WISP-2 (CCN5) and WISP-3(CCN6)^52^. Most of these proteins function to induce cell proliferation, extra-cellular matrix formation, cell adhesion and migration and their derailment eventually leads to angiogenesis and tumorigenesis^53^. CTGF has been implicated in a number of human cancers for its pivotal role in the induction of metastasis and invasion^17^,^54^. CTGF is a bad prognostic marker in GBM as the patients with high expression of CTGF have significantly less survival^55^. CTGF expression resulted in the induction of various anti-apoptotic proteins BCL-xl, Survivin and Flip in GBM, resulting in an aggressive phenotype as the cells became refractory to various chemotherapeutic agents and other adverse conditions^54^. CTGF expression also leads to the loss of IkB, thereby enhancing the NFkB activity in the nucleus^54^. CTGF was also found to play a prominent role in glioma cell invasion by activating ITGB1-TrkA-NFkB axis, wherein it resulted in the activation of ZEB-1 (a transcriptional repressor), which subsequently brought down the levels of e-cadherin and thereby enhanced migration and invasion^18^. Our findings also support the existing role of CTGF, as we observed that the expression of CTGF was high in various GBM data sets and the knock down of CTGF phenocopied the effects, as produced by Cbx7 re-introduction. Moreover, the exogenous expression of CTGF in Cbx7 stable background ensued in the rescue of the migratory potential of glioma cells. This establishes the fact that the loss of Cbx7 in GBM is potentially one of the mechanisms adopted by the cancer cell to increase CTGF levels and become more aggressive (Fig. 8). Our study also provides evidence that Cbx7 eventually exerts its oncosuppressive phenotype by abrogating the activity of SAPK/JNK, the same finding was also observed upon CTGF silencing. In addition to that, CTGF overexpression in the Cbx7 stable background brought back the levels of active SAPK/JNK. Similarly, the over-expression of constitutively active SAPK/JNK resulted in the reversal of the inhibition of migration in Cbx7 stable background. This demonstrates that an active SAPK/JNK pathway is necessary for glioma cell migration and can be targeted for chemotherapeutic purposes. So it could be deduced that Cbx7 regulates the activity of SAPK/JNK through CTGF modulation. Though there is minimal information known about the signalling mediated by CTGF, there are few reports which indicate that CTGF stimulates p42/44 mitogen activated protein kinase (p42/44 MAPK) and p38 MAPK in a human chondrosarcoma derived chondrocytic cell line and induces chondrocyte differentiation^56^. Also in human corneal epithelial cells CTGF has been shown to play a role in EMT via Ras/MEK/ERK signalling axis^57^. In yet another study, CTGF was found to increase phosphorylation of five phosphoproteins including SAPK/JNK^58^. Retrospectively, there are a number of reports which indicate the regulation of CTGF by SAPK/JNK signalling wherein CTGF has been shown to produce its effects in the presence of active SAPK/JNK pathway^59^,^60^.

In summary, this study indicates that Cbx7 hypermethylation is an important event in gliomagenesis and adds one more realm of misregulation to the malignant cell, in order to incapacitate the anti-tumorigenic Hippo pathway, thereby underscoring the importance of Cbx7 downregulation in GBM (Fig. 8). Also, the somatic and germline mutations rarely invade the hippo pathway, as neurofibromin (NF2) or merlin is the only upstream pathway gene which has been known to be mutated and is thus recognized as a bonafide tumour suppressor gene. This infers that this pathway is mostly inactivated by mechanisms resulting from molecular events other than the somatic mutations of the pathway genes, thereby intimating to the role of epigenetic modifiers like Cbx7, which are also circumvented in glioma. In other words Cbx7 augments the Hippo pathway by monitoring the YAP/TAZ dependent transcriptional activity which gets breached in the glioblastoma scenario.

## Materials and Methods

### Human tumour samples and biosafety clearance

The tumour samples used in the study were obtained from patients who were operated in Sri Sathya Sai Institute of Higher Medical Sciences (SSSIHMS) and National Institute of Mental Health and Neurosciences (NIMHANS), Bangalore, India. The non-tumour brain used as control, comprised of brain tissue (anterior temporal lobe) obtained during the surgery of intractable epilepsy cases. The tissues both tumour and control were snap-frozen in liquid nitrogen and stored at −80 °C and used for DNA/RNA isolation. This study has been scrutinized and approved by the ethics committee of NIMHANS (NIMHANS/IEC/No. RPA/060/05 dated 29.10.2005) and SSSIHMS (SSSIHMS/IEC/No. RPA/001/2005 dated 20.10.2005). An informed written consent was obtained from all patients prior to initiation of the study as per IEC guidelines and approval. A total of 118 GBM samples and 17 control brain samples were used in this study. Out of these samples, 82 GBMs and 8 control brain samples were used for quantitative RT-PCR validation of the Cbx7 gene, and 36 GBM and 9 control brain samples were used for the Infinium methylation array.

All methods and experimental protocols adopted in this study are in accordance with the guidelines approved by the Institutional biosafety clearance committee of Indian Institute of Science, Bangalore.

### Genomic DNA Extraction and Sodium Bisulphite Conversion

Genomic DNA was isolated from tumour tissues and control brain samples using QIAamp DNA minikit (Qiagen). DNA quality was assessed on a low per cent agarose gel and was quantified to be used for bisulphite conversion. DNA was subjected to bisulphite conversion using Premium Bisulphite Kit (Diagenode Inc. USA). 1 μg of purified genomic DNA with its volume made up to 20 μl was mixed with 130 μl of the conversion reagent in a PCR tube. The reaction mixture was heated at 98 °C for 8 minutes followed by incubation at 54 °C for 60 min and a final halt at 4 °C. The above mixture was overlaid onto 600 μl of binding buffer in a spin column. The spin column was centrifuged at 12000 rpm for 30 seconds and the flow through was discarded. 100 μl of wash buffer was added to the spin column having bound DNA and was centrifuged at 12000 rpm for 30 seconds. This was followed by the desulphonation of the bisulphite treated DNA. The desulphonation buffer was removed as flow through, by centrifugation at 12000 rpm for 30 seconds. Lastly the column was again washed with wash buffer and the bisulphite converted DNA was eluted with 20 μl of elution buffer. Eluted DNA was either used immediately for bisulphite sequencing or stored at −20 °C for later use.

### Methylation analysis

Infinium HumanMethylation450K BeadChip (Illumina, San Diego, CA) was used for the analysis of the tumour methylome. Infinium array examines 485,000 methylation sites per sample at single-nucleotide resolution covering around 99% of the RefSeq genes with an average of 17 CpG sites per gene. DNA methylation analysis was performed using 36 GBM and 9 control brain samples. The intensities obtained from the Infinium array were used to calculate the beta value, using the following formula: beta value = (signal intensity of M Probe)/[(signal intensity of M + U probes) +100].

### Cell lines and Plasmids

Glioma cell lines U87, U138, U251, U343, U373, LN229, LN18, T98G and A172 were grown in DMEM while SVG cells were grown in MEM. Most of the used cell lines were procured from ECACC. The medium was supplemented with 10% FBS, penicillin and streptomycin. Immortalized human astrocytes were a kind gift from late Dr. Abhijit Guha. pCMV-Entry, pCMV-Cbx7 and pCMV-CTGF were purchased from Origene, USA. pcDNA3-Myc-SAPKβ-MKK7 (SAPK/JNK-CA; constitutively active form of JNK/SAPK) was a kind gift from Dr. Jakob Troppmair^61^.

### Bisulphite Sequencing

Cbx7 gene was scanned for its CpG island in its promoter region using an online tool; MethPrimer (http://www.urogene.org/cgi-bin/methprimer/methprimer.cgi). The region for sequencing was chosen in such a way that it encompasses the specific CpG assayed in the methylation beadChip array (cg23124451). The region was amplified by PCR, wherein the reaction mixture contained 100 nM of each primer, 200 ng of bisulphite-converted DNA, 2U of MyFi DNA Polymerase (Bioline, USA), 5X MyFi reaction buffer and the final volume was made upto 25 μl. The PCR products were cloned using the pGEM-T easy vector system (Promega) which is based on TA cloning principle. The transformants were selected on an LB ampicillin plate. True positives carrying the region of interest were screened by means of colony PCR using M13 primer pair. Plasmid DNA was prepared from at least 10 true clones representing one particular DNA sample, using MDI mini prep kit. The purified plasmid DNA was subjected to sequencing by M13 reverse primer. Sequences thus obtained were analysed for methylation pattern by aligning them to the genomic DNA sequence using the bisulphite sequencing web-tool QUMA software and the degree of methylation was represented as a lolli-pop grid.

### 5-Aza-2’-deoxycytidine treatment

Glioma cell lines were seeded at a density of 0.8 * 10^6^ per 60 mm dish. 24 hours after seeding, the cells were treated with 10, 20 and 50 μM of 5-aza-2′-deoxycytidine for 3 days and 5 days. The media was replaced every 24 hours with the fresh addition of the 5-aza-2′-deoxycytidine. Total RNA was isolated at respective time points and used to assay the levels of Cbx7 by qRT-PCR.

### Stable Cell Line Generation

In order to generate the stables of Cbx7 in glioma cell lines, cells (0.5 * 10^6^) were plated in 35 mm dish and transfected with the respective plasmids. This was followed by selection of stables after 48 hours of transfection, by allowing the cells to grow in presence of G418 (800–1200 μg) for 2 weeks. The representative stables were also harvested for RNA to confirm the overexpression of Cbx7.

### Colony suppression assay

Glioma cell lines were plated in a 6-well cluster plate (0.5 * 10^6^) and 24 hours later transfected with the control vector or Cbx7 overexpression vector using Lipofectamine 2000. Cells were subjected to G418 (800–1200 μg/ml) selection after 48 hours of transfection. Cells were incubated at 37 °C for 3 weeks with growth media being replaced every third day. Colonies were fixed in chilled methanol for 30 minutes followed by staining with 0.5% crystal violet for another 30 minutes. Stable Cbx7 cells and their respective vector control cells were counted and then seeded at a density of 2500–5000 cells per well of a 6-well cluster plate, followed by similar incubation and staining.

### Wound healing scratch assay

Cbx7 stables and their respective control cells were seeded in 6-well cluster plate (1.2 * 10^6^ cells/well) with 1.5 ml of complete DMEM. At 24 hours, the monolayers were mechanically disrupted with a sterile toothpick to produce a clean uniform scratch. The assay was performed in duplicates. The wells were photographed every 12 h to monitor the closing of the wound.

### Cell migration and invasion

Migration of cancer cells was assayed in 24-well Boyden chambers with 8-μm pore size polycarbonate membranes (BD Biosciences, San Diego, USA). For invasion assay, the membranes precoated with matrigel were used (BD Biosciences, San Diego, USA). Cells (5 × 10^4^) were resuspended in 500 μl serum-free medium and placed in the upper chamber, and the lower chamber was filled with 750 μl medium with 10% FBS. Cells were incubated for 22 hours and after incubation, the cells remaining on the upper surface of the membrane were removed by wiping with a wet cotton bud. The cells on the lower surface of the membrane were fixed in chilled methanol and stained with crystal violet and counted under a light microscope.

### Luciferase Assay

To assess the effect of Cbx7 on the promoter activity of CTGF or other targets of YAP/TAZ, cells were plated in a 6-well cluster plate and co-transfected with the pCMV-Entry and pCMV-Cbx7 along with CTGF promoter vector (CTGF-Luc/FL, CTGF-Luc/M) or TEAD-luc vector (8xGTIIC-luciferase; Addgene) and pCMV-beta gal using lipofectamine following manufacturer’s instructions. After 48 hours of plasmid transfection, cell extracts were made in reporter lysis buffer (Promega). Protein concentrations of the cell lysates were measured by Bradford assay reagent (Bio-Rad). 10 μg equivalent protein was mixed with 30 μl of luciferase assay reagent (LAR) to determine the luciferase activity and these values were normalised by beta galactosidase activity units.

### Western blot analysis

Cells were lysed using RIPA (RadioImmunoPrecipitation Assay) buffer. Protein lysates were quantified by Bradford assay reagent (Bio-Rad) before subjecting the same to immunoblotting. The proteins were separated on 10 or 12% SDS-PAGE and then transferred to PVDF membrane (Merck Millipore). Samples belonging to a particular experiment were run in a same gel under same experimental conditions. For CTGF expression analysis, the conditioned media (culture media of an experiment) was collected (5 ml) and concentrated in Amicon centrifugal filter device, which has cut-off of 3 kDa. Equal volumes of the concentrated conditioned media were loaded in a polyacrylamide gel. The antibodies that were used in the study include: Anti-DDK antibody (Origene; cat # TA50011), Phospho-Akt (Ser473) (Cell Signalling technology; cat # 4060), Akt (Cell Signalling technology; cat # 9272), anti-beta Actin HRP (Sigma; cat # A3854), phospho-SAPK/JNK (Cell Signalling technology; cat # 9251), SAPK/JNK (Cell Signalling technology; cat # 9258), Phospho-p44/42 MAPK (Erk1/2) (Cell Signalling technology; cat # 9101), p44/42 MAPK (Erk1/2) (Cell Signalling technology; cat # 9102), anti-alpha tubulin (Merck Millipore; cat # CP06), caspase-3 (Cell Signalling technology; cat # 9661), anti-Cbx7 (Abcam; cat # ab21873).

### siRNA transfection

CTGF siRNA pool was purchased from Dharmacon (USA) and transfected (100 nM) using Dharmafect1 transfection reagent. 48 hours after transfection, cell were trypsinized, counted and re-plated for experiments. Remaining cells were used for RNA isolation to perform qRT-PCR.

### Gene ontology analysis

Gene ontology mining and pathway analysis was carried out using DAVID (https://david.ncifcrf.gov/) Bioinformatics Resources tool.

### GSEA analysis

Enrichment of signalling pathway in the differentially expressed genes was carried out by using Gene Set Enrichment Analysis (GSEA) tool. YAP and TAZ target genes^14^,^15^,^16^ were provided as gene sets to perform GSEA.

## Additional Information

**How to cite this article**: Nawaz, Z. *et al*. Cbx7 is epigenetically silenced in glioblastoma and inhibits cell migration by targeting YAP/TAZ-dependent transcription. *Sci. Rep.* **6**, 27753; doi: 10.1038/srep27753 (2016).

## Supplementary Material

Supplementary Information

Supplementary table ST1

Supplementary table ST2

Supplementary table ST3

Supplementary table ST4

Supplementary table ST5

Supplementary table ST6

Supplementary table ST7

Supplementary table ST8
