# Supplementary material for: Cbx7 is epigenetically silenced in glioblastoma and inhibits cell migration by targeting YAP/TAZ-dependent transcription
Source: Sci Rep. 2016 Jun 13;6:27753. doi: 10.1038/srep27753 (PMC4904208; doi:10.1038/srep27753)
Supplement: Supplementary Information [file srep27753-s1.pdf]

Cbx7 is epigenetically silenced in glioblastoma and inhibits cell migration by targeting YAP/TAZ-dependent transcription

Zahid Nawaz[a], Vikas Patil[a], Anjali Arora[a], Alangar S Hegde[b], Arimappamagan Arivazhagan[c], Vani Santosh[d] and Kumaravel Somasundaram[a,*]

Department of Microbiology and Cell Biology[a], Indian Institute of Science, Bangalore 560012; Department of Neurosurgery[b], Sri Satya Sai Institute of Higher Medical Sciences, Bangalore 560066; Departments of Neurosurgery[c] and Neuropathology[d], National Institute of Mental Health and Neuro Sciences, Bangalore 560029

[*]- Corresponding author
Tel: 91 80 23607171
Fax: 91 80 23602697
Email: skumar@mcbl.iisc.ernet.in; ksomasundaram1@gmail.com

Additional Supplementary methods

Other Data sets

In order to compare and corroborate the methylation and expression of Cbx7 and other genes evaluated in the study, the fold change and beta values were further derived from various publicly available data portals, including The Cancer Genome Atlas (TCGA) dataset (Agilent and Affymetrix platforms), REMBRANDT dataset and GSE22867 dataset.

RNA extraction, cDNA conversion and Quantitative RT-PCR analysis

Total cellular RNA was isolated using Trizol reagent (sigma). RNA thus isolated was analysed for its purity and integrity by Nano-drop and gel electrophoresis. 2 µg of total RNA was used for cDNA conversion using High capacity cDNA reverse transcription kit the (applied biosystems, USA) according to the manufacturer's protocol. cDNA generated was diluted with nuclease free water in the ratio of 1: 10 such that the final concentration of the cDNA was 10 ng/ul. Real-time PCR was done using the ABI PRISM 7900 HT Sequence Detection System (Life technologies, USA) under default conditions: 95°C for 15 minutes, 40 cycles of 95°C for 20 seconds, 60°C for 25 seconds and 72°C for 30 seconds. Expression was analyzed using GAPDH, 18S RNA and RPL35a as a reference gene and the ddCt method.

Transcriptome analysis by RNA sequencing

The RNA samples were assessed for quality and quantity using Agilent's Bioanalyser. 1 ug of RNA from each sample was used for library preparation. The library for sequencing was prepared using TrueSeq RNA sample preparation kit as per the manufacturer's guidelines (Cat # RS-122- 2001). The library was then re-quantified using Agilent's Bioanalyser as well as real-time qPCR. 120 ul (10pM) of each sample library was taken, the strands were denatured and finally subjected to cluster generation on the flow-cell in the c-Bot system using TruSeq PE Cluster kit (Cat # PE-401-3001). The flow cell was finally subjected to two rounds of sequencing (Read 1 and Read 2) and the results were obtained as

intensity files. Sequencing was conducted on Illumina HiScanSQ using Truseq SBS V3 technology for 50 base pair paired-end reads RNA sequencing (Cat # FC-401-3002). Raw reads obtained were mapped to the human reference genome (hg19) using TopHat (version 2.0.10). The alignment files were thereafter subjected to Cufflink (version 2.2.0) to generate a transcriptome assembly. Each of the transcriptome assemblies was merged using Cuffmerge utility, to generate the final transcriptome assembly. The transcriptome assembly thereby generated and alignment file were analysed through Cuffquant utility to quantify gene and transcript expression. Normalization of quantified gene and transcript expression was carried out using Cuffnorm utility. Differentially expressed genes were identified using Cuffdiff utility and those having fold change above +/- 1.5 with FDR-adjusted p-value <0.05 were considered.

Supplementary figure legends

Figure S1,

A, B, Expression (transcript) levels of different chromobox proteins in GBM with **A,** comprising of the heterochromatin proteins and **B,** comprising of the Polycomb group proteins.

C, D, Methylation profile of the chromobox proteins with **C,** depicting the beta values of the heterochromatin genes and **D**, comprising of the Polycomb group genes. Each gene has two probes in the methylation array and so is represented by two beta values.

Figure S2, A, B and C, Glioma cell lines U373, U87 and U343 were transfected with control vector or pCMV-Cbx7 and selected for G418-resistant colonies for a period of 2-3 weeks. The colonies were stained with crystal violet and the mean colony counts are displayed as percentage of control vector. The t-test was performed between control vector and cbx7 over-expression condition using student's t-test and the *p* value symbols are indicated, (**) $p \leq 0.01$ and (***) $p \leq 0.001$.

Figure S3, A, B and C, Heat maps indicating the set of genes, targets of YAP/TAZ mediated transcription being downregulated in Cbx7 overexpression state and a majority of them being upregulated in GBM. Genes were derived from three independent studies.

Figure S4,

A, Total cellular protein was extracted from LN229/VC and LN229/Cbx7 stable cells and the expression levels of indicated proteins was assayed using Western blot, *- refers to a non-specific band obtained with anti-Cbx7 antibody.

B, **C**, Total cellular protein was extracted from LN229 and A172 cell lines after treatment with SP700125 (50μM) and the expression level of indicated proteins was assayed using Western blot.

Supplementary figures

Supplementary Figure S1; Nawaz et.al 2015

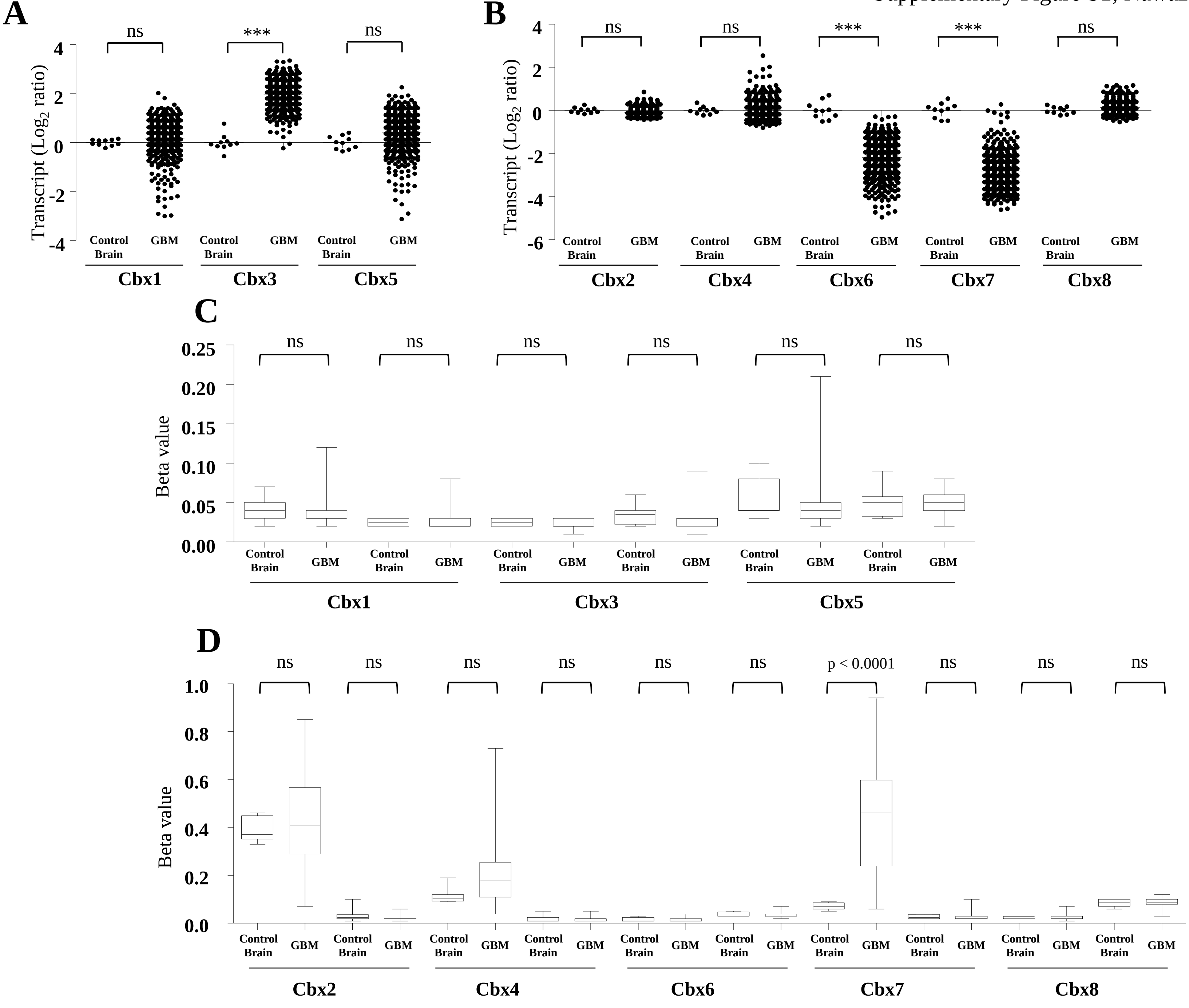

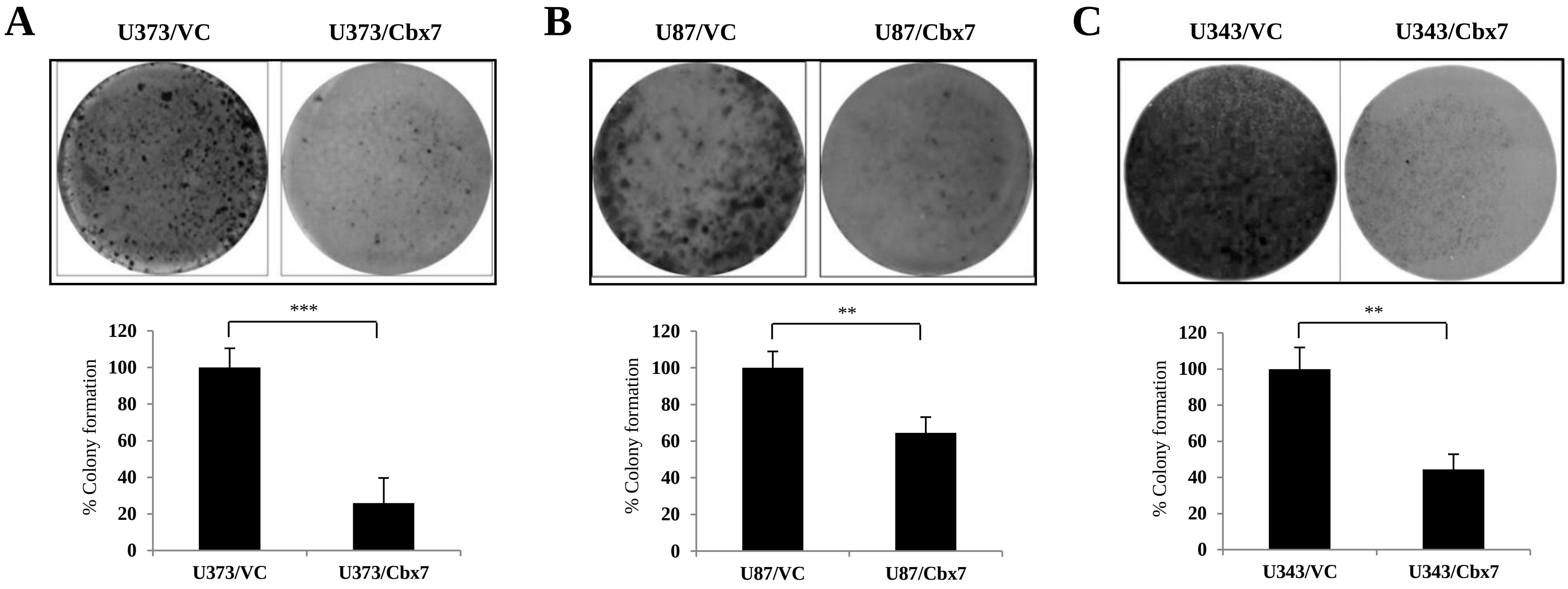
A
U373/VC
U373/Cbx7
***
% Colony formation
120
100
80
60
40
20
0
U373/VC
U373/Cbx7
B
U87/VC
U87/Cbx7
**
% Colony formation
120
100
80
60
40
20
0
U87/VC
U87/Cbx7
C
U343/VC
U343/Cbx7
**
% Colony formation
120
100
80
60
40
20
0
U343/VC
U343/Cbx7

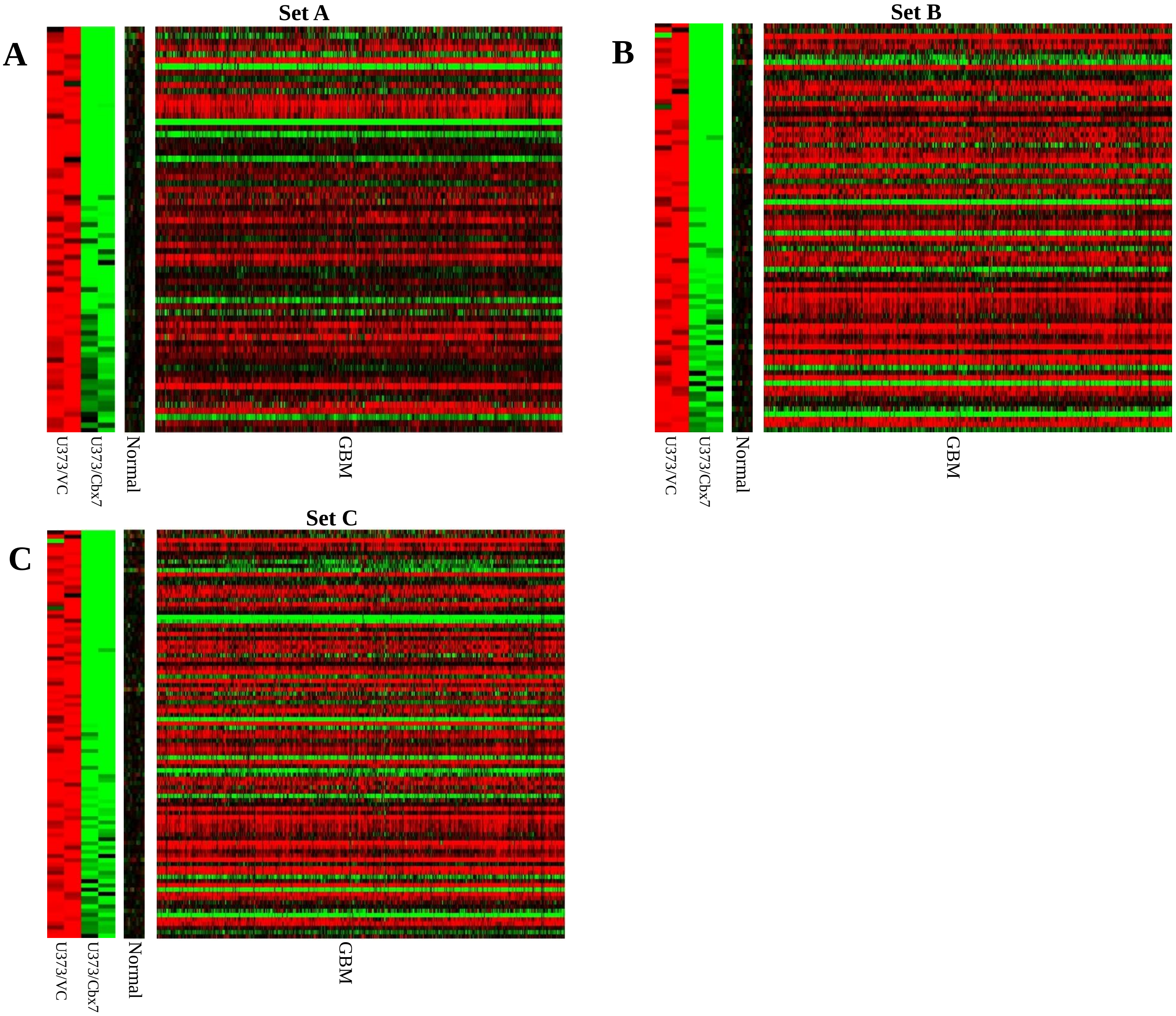

Supplementary Figure S3; Nawaz et.al 2015

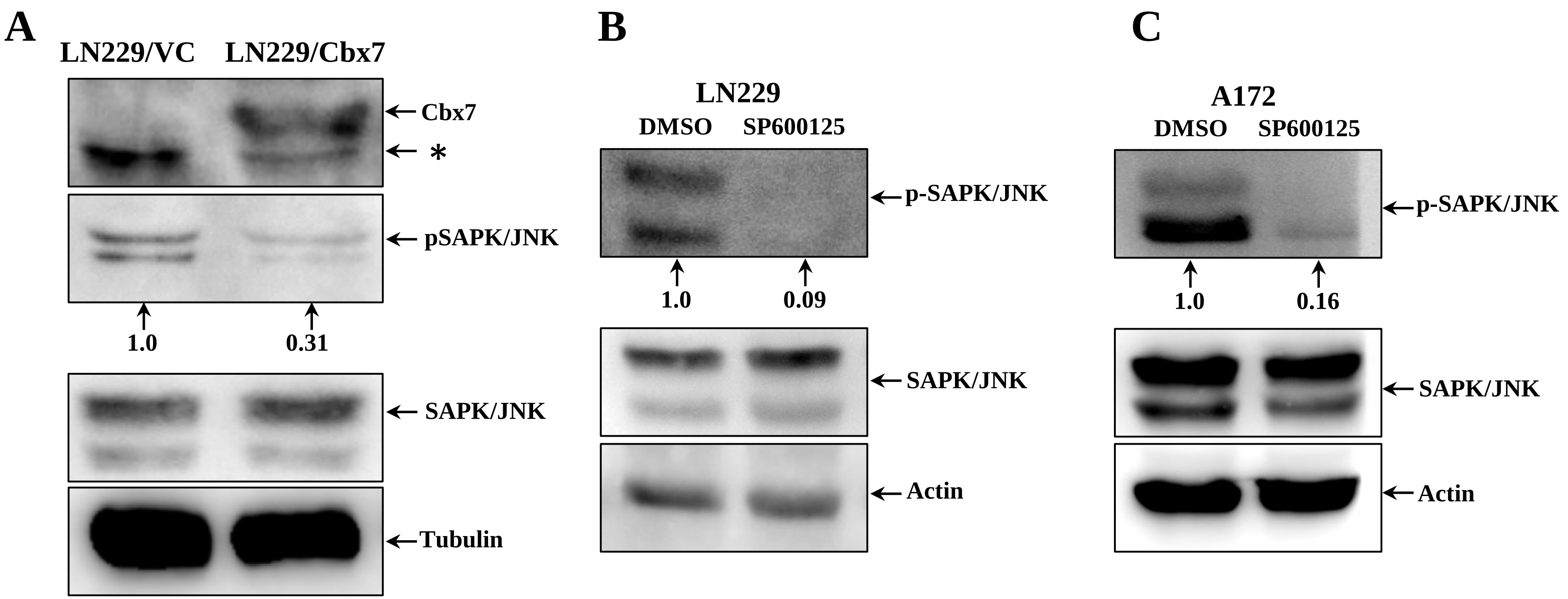
A
LN229/VC
LN229/Cbx7
Cbx7
*
pSAPK/JNK
1.0
0.31
SAPK/JNK
Tubulin
B
LN229
DMSO
SP600125
p-SAPK/JNK
1.0
0.09
SAPK/JNK
Actin
C
A172
DMSO
SP600125
p-SAPK/JNK
1.0
0.16
SAPK/JNK
Actin
